# Antimicrobial Peptides in Fish: Mechanisms of Action and Applications in Aquaculture

**DOI:** 10.3390/biology15100790

**Published:** 2026-05-15

**Authors:** Fan Zhou, Leyi Zhou, Pengfei Wang, Mariano Elisio, Sally Salaah, Bakhtiyor Karimov, Quanquan Cao

**Affiliations:** 1 Fisheries College, Sichuan Agricultural University, Chengdu 611130, China; 2 Institute of Hydrobiology, Zhejiang Academic of Agricultural Sciences, Hangzhou 310021, China; 3 Instituto Nacional de Investigacion y Desarrollo Pesquero, Mar del Plata B7602HSA, Provincia de Buenos Aires, Argentina; 4 National Institute of Oceanography and Fisheries (NIOF), Cairo 11796, Egypt; 5 Tashkent Institute of Irrigation and Agricultural Mechanization Engineers, National Research University, Tashkent 100000, Uzbekistan

**Keywords:** antimicrobial peptides, aquaculture, immune regulation, antibiotic alternatives, sustainable development

## Abstract

With the rapid development of global aquaculture, the frequent occurrence of fish diseases has had a serious impact on the efficiency of aquaculture and the ecological environment. Antimicrobial peptides, as a kind of natural immune active substance existing in organisms, participate in innate immunity and adaptive immunity. Due to their extensive antibacterial properties and low toxicity, they have gradually become a hot topic in scientific research. This article reviews the classification, tissue distribution, mechanism of action, extraction, and synthesis techniques of antimicrobial peptides (AMPs) derived from fish, as well as their applications in disease prevention in aquaculture, product preservation, and antibiotic substitution. Although antimicrobial peptides are expected to become alternatives to antibiotics, challenges such as environmental stability, production costs, and regulatory frameworks remain to be addressed. This article holds that antimicrobial peptides derived from fish are a feasible strategy for sustainable aquaculture. The future development direction lies in biotechnology-driven optimization, carrier innovation, and combined application with traditional antibiotics.

## Simple Summary

This study systematically reviews fish-derived AMPs’ classification, tissue distribution, and extraction/synthesis techniques with respective pros and cons. It elucidates fish AMPs’ dual mechanisms: direct antibacterial and immune regulation. It highlights fish AMPs’ aquaculture applications: disease control, aquatic product preservation, and low-resistance antibiotic alternatives.

## 1. Introduction

As a vital component of the global food supply chain, aquaculture provides a stable and efficient source of animal protein and is expected to double its production by 2050 [1,2,3]. However, for disease outbreaks in intensive aquaculture systems, bacterial pathogens represent the largest proportion of disease-causing agents in aquaculture, with common genera including *Vibrio*, *Pseudomonas*, *Aeromonas*, *Nocardia*, *Edwardsiella*, and *Streptococcus* contributing to significant health challenges in fish [4]. Typically, antibiotics are used globally as medications or feed additives in aquaculture to prevent and control diseases. To some extent, antibiotics provide fish with a certain level of protection against infectious diseases. Scientific research shows that the long-term abuse of antibiotics not only causes drug residue problems but also leads to the development of antibiotic-resistant bacteria (ARBs) and resistance genes (ARGs) [5,6]. According to research, up to 75% of antibiotics in feed are either not fully utilized or excreted into the aquaculture environment. These inadequately treated antibiotic residues often directly enter the surrounding ecosystem, posing serious ecological risks [7].

With the continuous increase in ARB and ARG, it is particularly urgent to find new antibacterial agents to replace antibiotics. To date, antimicrobial peptides (AMPs) have attracted global attention due to their excellent biocompatibility, low residue levels, and effectiveness against antibiotic-resistant bacteria [8]. Their potential in combating drug-resistant infections lies in their unique bactericidal mechanisms, making them key effector molecules in the host immune system of organisms. Specifically, the advantages of AMPs include a low likelihood of inducing pathogen resistance, a broad antimicrobial spectrum, and distinct modes of action. AMPs exert their antimicrobial effects through diverse mechanisms. A well-characterized mechanism involves binding to the negatively charged regions of bacterial cell membranes, leading to membrane disruption, osmotic imbalance, and ultimately cell lysis and death. In addition, many AMPs act through non-membrane-targeting mechanisms, including the inhibition of cell wall synthesis, interference with nucleic acid or protein synthesis, suppression of efflux pump activity, and disruption of key intracellular metabolic pathways [9].

Antimicrobial peptides derived from fish can be obtained in a variety of ways: they are expressed in fish skin, gills, guts, and immune organs and are secreted to the mucosal surface to form the first barrier against pathogens [10]. They can also be extracted directly from fish tissue or by processing by-products by acid-base hydrolysis, enzymatic hydrolysis, or solvent extraction [11]. In addition, AMPs are also endogenously synthesized when fish respond to infection or stress and can even be recovered from aquaculture water as excretion or secretion products, providing a new method for sustainable utilization [12,13]. Fish-derived AMPs have gradually developed into practical health products in aquaculture. As feed additives, they can improve growth performance, enhance immune response, and reduce intestinal pathogen colonization [14]. Used as surface coatings and preservatives, they can inhibit spoilage microorganisms and extend the cold chain storage period [15]. They can also be used as an active packaging material to embed degradable film to achieve slow-release antibacterial effects [16]. These applications fully reflect the potential of fish-derived AMPs to transform from basic research to industrial practice and provide an innovative product development path for aquatic animal health management.

This paper mainly summarizes the classification, mechanism of action and application prospect of fish-derived antimicrobial peptides in aquaculture, aiming to provide a theoretical basis for the in-depth research and industrial application of fish antimicrobial peptides.

## 2. Overview of Antimicrobial Peptides in Fish

### 2.1. Definition and Characteristics

Antimicrobial peptides (AMPs), also known as host defense peptides, are small molecule peptides synthesized by ribosomes. They typically consist of 7–100 amino acids, have a molecular weight below 10 kDa, and carry a net positive charge of +2 to +11. The positive charge is attributed to the presence of specific amino acids, such as arginine (Arg), lysine (Lys), and so on. These types of active peptides not only have the ability to directly inhibit or kill microorganisms but also play an important role in immune regulation and the host’s innate and adaptive immune defense system [17,18]. To date, the Antimicrobial Peptide Database (APD) has recorded a total of 22,724 monomeric peptides, 403 polymeric peptides, and 236 peptides as monomers, dimers, and higher-order oligomers, respectively [19]. The amphiphilicity of AMPs refers to the structural characteristics of both hydrophobic and hydrophilic amino acid regions in their molecules. This structure enables AMPs to be stably soluble in aqueous media and effectively embedded in lipid environments (such as cell membranes) [20,21]. Amphiphilic balance is key to the function of AMPs, which affects the binding ability of peptides with target membranes and membrane destruction efficiency. Studies have shown that moderate regulation of amphiphilicity can enhance antibacterial activity and reduce hemolytic toxicity to red blood cells [22].

### 2.2. The Tissue Distribution Characteristics

AMPs, as important effector molecules of the immune system that function in both innate and adaptive immunity, have been successfully isolated and identified in various organisms including microorganisms, vertebrates (fish, amphibians, birds, mammals), and invertebrates [23]. These peptides play a crucial role in the host’s non-specific immune defense mechanism, forming the first biological barrier against pathogen invasion [24]. The latest research shows that the constitutive expression of AMPs genes can be detected in different tissues through quantitative polymerase chain reaction (PCR) technology, but there are significant tissue-specific differences in their expression levels [25]. As a representative of aquatic vertebrates, the distribution and function of AMPs in the immune systems, skin mucus layers, gills, and intestines of fish have become a key entry point in analyzing the innate immune adaptation mechanism of aquatic organisms.

In the immune system of fish, the mucous layer of the skin contains abundant immune-active substances [26]. Goblet cells beneath the epithelial layer of fish are responsible for mucus secretion and contain various immune effector molecules, including lectins, mucins, and peptides with antibacterial activity [27]. Among them, the antimicrobial peptide family is particularly diverse, mainly including subtypes such as β-defensin and hepcidin, and these peptides exhibit significant inhibitory activity against pathogens such as bacteria, fungi, and viruses [28]. For example, the paradaxin secreted by the mucous glands of *Pardachirus marmoratus* has potent antibacterial and hemolytic activity [29]. Misgurin derived from loaches maintains high antibacterial activity while significantly reducing hemolytic toxicity [30]. The β- defensin-like protein 1 isolated from the epidermal mucus of carp (*Cyprinus carpio* L.) using an improved acetone precipitation method showed excellent in vitro antibacterial properties [31]; the novel antibacterial protein identified in the epidermis of catfish (*Clarias batrachus*) exhibited unique molecular characteristics and broad-spectrum antibacterial activity [32]. Immune active substances such as antimicrobial peptide LEAP-1 were also detected in the skin secretions of carp [33].

As a multifunctional organ, fish gills, while fulfilling respiratory functions, prominently serve as a crucial mucosal immune barrier by secreting AMPs [34]. A typical example is the study by Alessi et al., which found the expression of AMPs piscidin 1 in gills mast cells of giant mudskipper (*Periophthalmodon schlosseri*) [35]. This discovery not only confirms the key role of AMPs in the immune defense of teleost fish but also provides new theoretical basis for a deeper understanding of the synergistic defense mechanism of the fish immune system.

The intestinal immune system of fish uses its gut-associated lymphoid tissue (GALT) to secrete various AMPs to resist pathogen infections, such as the piscidins AMPs widely present in the gut and skin of perciform fish (such as hybrid striped bass and European sea bass), which exhibit significant broad-spectrum antibacterial activity against pathogenic bacteria such as *Vibrio parahaemolyticus* and *Aeromonas hydrophila*, as well as fungi and parasites [36]. The homeostasis of gut microbiota plays a key role in fish mucosal immunity [37,38]. Unlike antibiotic treatment, which can easily lead to microbial imbalance, endogenous AMPs in the gastrointestinal tract provide a better option for regulating and rebuilding gut microbiota [39]. For instance, the dietary supplementation of 400–800 mg/kg AMPs can significantly improve the intestinal morphology and structure of grass carp and enhance its digestive function, antioxidant capacity, and immune response while optimizing the composition of intestinal microbiota [40]. In addition, some AMPs can be synthesized and secreted by immune cells such as macrophages and neutrophils, further enhancing their important role in the fish immune defense network.

### 2.3. Extraction and Synthesis Techniques of AMPs in Fish

**Figure 1:**
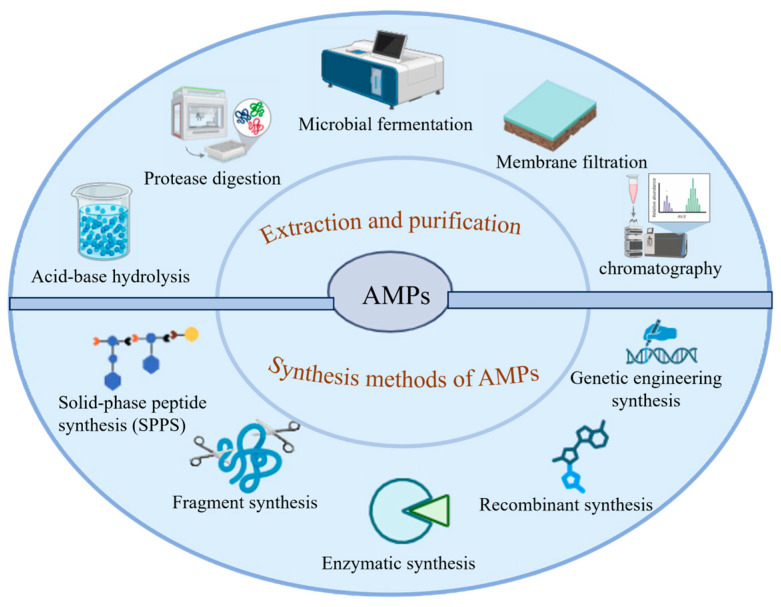
This diagram illustrates the technologies related to Antimicrobial Peptides (AMPs), covering protease digestion, acid-base hydrolysis, microbial fermentation, membrane filtration, and chromatography in terms of extraction and purification, as well as solid-phase peptide synthesis, fragment synthesis, enzymatic synthesis, recombinant synthesis, and genetic engineering synthesis in terms of synthesis methods. Created in by Fan Zhou (2025) https://BioRender.com (accessed on 10 May 2025).

There are currently many methods for extracting and synthesizing AMPs. Figure 1 shows the extraction, purification, and synthesis methods of antimicrobial peptides. Regarding the extraction, purification, and synthesis methods of AMPs, existing research has provided multiple technical pathways with their own advantages and disadvantages. In terms of extraction and purification, it mainly includes acid-base hydrolysis, protease digestion, microbial fermentation, membrane filtration, and chromatography. In terms of synthesis methods, there are mainly solid-phase peptide synthesis (SPPS), fragment synthesis, enzymatic synthesis, recombinant synthesis, and genetic engineering synthesis.

#### 2.3.1. Extraction and Purification Techniques

Acid base hydrolysis uses acid or base treatment to cleave amino bonds in protein sequences, and H^+^ or OH^−^ attacks the carbonyl carbon of peptide bonds to form tetrahedral intermediates, which eventually leads to the breaking of C-N bonds [41]. For instance, acid extraction followed by enzymatic hydrolysis has been employed to obtain histone-derived antimicrobial peptides from *Atlantic salmon* testis [42], as well as bioactive protein hydrolysates from grass carp (*Ctenopharyngodon idella*) scales [43].

Protease digestion uses specific proteases, such as trypsin, pepsin, papain, and subtilisin, to selectively cleave specific amino acid sites under mild conditions [44,45,46]. Trypsin cleaves the carboxyl terminus of lysine and arginine; Pepsin prefers the amino terminus of aromatic amino acids [47]. By controlling the ratio of the enzyme to the substrate, hydrolysis time, and termination reaction, the active peptide with a specific molecular weight range can be obtained [48]. Alkaline pre-treatment is often employed prior to enzymatic hydrolysis to remove non-collagenous proteins. The researchers demonstrated that sequential treatment with 0.3 M NaOH for 4 h followed by Protamex^®^ enzymatic hydrolysis effectively extracted bioactive collagen peptides from snakehead fish (*Channa striatus*) waste skin [49].

Microbial fermentation uses microorganisms, including proteases secreted by prokaryotic or eukaryotic expression systems during growth and metabolism, to hydrolyze protein substrates and release active peptides [50]. Commonly used bacteria include lactic acid bacteria, bacillus, yeast, and mold [51]. This method can produce transgenic antimicrobial peptides on a large scale [52]. In addition, microorganisms themselves can also serve as a source of AMPS, such as bacteriocins [53].

Membrane filtration is a pressure-driven membrane separation technology based on molecular weight [54]. Ultrafiltration membrane (1–100 kDa) was used for primary separation, and nanofiltration membrane (<1 kDa) was used for fine fractionation [55]. Molecules in the solution pass through the membrane pore under pressure, molecules smaller than the molecular weight cut-off pass through the filter membrane, and the rest are cut off. This process does not require heating and chemical reagents and can maintain the bioactivity of the peptide [56]. Membrane filtration is an effective method for extracting antimicrobial peptides from various fish [57,58,59]. The optimization of membrane parameters including molecular weight cut-off (MWCO), membrane material, and surface charge is essential for the efficient purification of AMPs [60]. In the purification of fish antimicrobial peptides, the choice of MWCO needs to accurately match the molecular weight distribution of the target peptide [61]. The physical and chemical properties of membrane materials directly affect the adsorption behavior, flux stability, and cleaning and regeneration efficiency of peptides. Commonly used membrane materials include polyethersulfone (PES), polysulfone, and regenerated cellulose [62]. Most fish antimicrobial peptides have a net positive charge, and the negatively charged membrane is more conducive to the permeation of cationic peptides [63].

Chromatography uses the differential interaction between the peptide and stationary phase to achieve separation [64]. Commonly used chromatographic techniques mainly include ion exchange chromatography (IEC), gel filtration chromatography (GFC), reverse phase high-performance liquid chromatography (RP-HPLC), and hydrophobic interaction chromatography (HIC) [65]. Generally, it is necessary to use multidimensional chromatography in combination and then perform crude separation and purification in order to finally obtain high-purity active peptides [66]. Table 1 summarizes the extraction and purification methods of antimicrobial peptides and their advantages and disadvantages.

#### 2.3.2. Synthesis Techniques

Solid phase peptide synthesis (SPPS) is to connect the peptide chain to the solid-phase carrier step-by-step, obtain the target peptide resin through continuous amino acid coupling and deprotection reactions, and then release the target peptide from the solid-phase carrier to obtain the target peptide [73]. It is mainly used to verify the activity and optimize the structure of antibacterial peptides in fish [74]. It can accurately synthesize natural or computer-designed peptides and quickly screen out candidate molecules with stronger activity, lower toxicity, and better stability through amino acid replacement, truncation, dimerization, C-terminal amidation, and other modifications [75,76]. For example, African catfish nacap-ii, sturgeon testis neopeptide, and European sea bass β-defensin have all been synthesized and verified through SPPS for antibacterial, antiviral, or immunomodulatory functions [77,78,79].

Fragment synthesis is a method for splitting the target long peptide into multiple short peptide fragments, which are obtained by solid-phase synthesis or liquid-phase synthesis and then assembled into a complete peptide chain by chemical or enzymatic connection [80,81]. For example, the penaeidin class 4 antimicrobial peptide of white shrimp (*Litopenaeus setiferus*) was chemically synthesized from two domains by natural chemical ligation [82].

Enzymatic synthesis uses proteases to catalyze the reverse reaction under non-physiological conditions. By optimizing the reaction conditions, proteases can catalyze the condensation reaction between acyl donors and acyl acceptors to form new peptide bonds [83]. Research is exploring the use of isopentenyl transferase and other site-specific modifications of existing antibacterial peptides, which can significantly enhance their membrane penetration ability and antibacterial efficacy. Although it has not been directly applied to fish antimicrobial peptides, it provides a new direction for functional optimization [84].

The principle of recombinant synthesis is to clone the gene encoding the target amps into the expression vector, express it in the host, and then purify it by affinity chromatography [85]. The prokaryotic expression systems used for the synthesis of antimicrobial peptides mainly include the *Escherichia coli* expression system, *Bacillus subtilis* expression system, and *Bacillus licheniformis* expression system [86,87,88]. The *Escherichia coli* expression system is superior to the prokaryotic expression system, which can be divided into three types according to the host: the animal expression system, plant expression system, and yeast expression system [89]. Among them, the yeast expression system is the most widely used. In order to improve expression efficiency, the fusion expression strategy is often used. The fusion of antimicrobial peptides and chaperones can protect host cells from antimicrobial peptide toxicity and promote soluble expression. After expression, the fusion tag is excised by protease to release the target peptide [90]. A key factor to consider for the recombinant production of antimicrobial peptides is the potential contamination of endotoxin when using the *E. coli* expression system [91]. Common endotoxin removal strategies include (i) upstream control: reducing endotoxin production from the source, and preferentially using gram-positive bacteria or endotoxin-free engineered strains; (ii) downstream purification: separation and removal of endotoxin, including affinity chromatography, membrane filtration and ultrafiltration, and precipitation; (iii) degradation: damaging the structure of endotoxin, including high-temperature dry baking, acid base hydrolysis, and endonuclease degradation [92].

Genetic engineering synthesis is the comprehensive application of molecular biology, bioinformatics, and computational biology technology to rationally design or direct the evolution of AMPS coding genes to obtain improved peptide sequences and efficient expression systems [93,94]. These key technologies include codon optimization, tandem multimer expression, gene editing (CRISPR), and artificial intelligence-aided design [95,96,97,98]. Table 2 summarizes the synthetic methods of antimicrobial peptides and their advantages and disadvantages.

### 2.4. Types of AMPs in Fish

AMPs derived from fish can be systematically classified into five major groups based on their molecular characteristics: LEAPs, β-defensins, histone derived peptides, cathelicidins, and piscidins (unique to fish but homologous to sericin) [104]. According to the function of AMPs, AMPs can be divided into four categories: antimicrobial peptides, antifungal peptides, antiviral peptides, and antiparasitic peptides. Based on the characteristics of their secondary structure, AMPs can be divided into five categories: α- helical conformation, β- folded conformation, α-/β- mixed conformation, linear structure, and cyclic/complex topological structure [105,106].

Liver-expressed antimicrobial peptides (LEAPs), also known as hepcidin, as the first blood-derived antimicrobial peptide that has been intensively studied, plays an important role in the center of innate immune defense and the regulation of iron metabolism [107]. Its molecular structure is composed of three typical regions: signal peptide, propeptide, and mature peptide, which have different functions. The signal peptide sequence mediates the directional transport of th4 endoplasmic reticulum, the propeptide region maintains the stability of the protein structure, and the mature peptide exerts biological activities [108].

The LEAP family includes LEAP-1 and LEAP-2, which both include a typical three-domain architecture in terms of structural composition [109,110]. LEAP-1 is widely believed to be involved in innate immunity in fish. The difference between them is reflected in that LEAP-1 contains eight highly conserved cysteine amino acid residues, which can form four pairs of intramolecular disulfide bonds, while LEAP-2 only retains four cysteines, forming two characteristic disulfide bond pairs [111]. LEAPs do not kill microbial cells by forming pores on bacterial membranes [112]. Hepcidin belongs to the cysteine-rich liver-specific expression protein family, and there are subtypes such as LEAP-1 and LEAP-2a/b/c in rainbow trout and grass carp, which are mainly specifically expressed in the liver [113]. Hepcidin, as a core regulator of iron homeostasis, maintains iron homeostasis by precisely regulating the circulatory system and intracellular iron concentration [114]. Hepcidin ferroportin signal interaction regulates the related signaling pathways of iron metabolism [115]. Studies have shown that hepcidin in fish exhibits significant broad-spectrum antimicrobial activity, including the property of inhibiting bacterial and viral infections [116]. It is worth noting that hepcidin in fish has a significant inhibitory effect on aquatic pathogens such as *Aeromonas hydrophila* and can reduce the incidence of bacterial diseases in grass carp [117]. Adding antimicrobial peptides to grass carp feed can improve feed conversion, promote growth performance, and enhance immune function [118].

β-defensins represent a class of antibacterial peptides with a characteristic β-sheet structure, and the stability of their three-dimensional structure depends on the intramolecular disulfide bond network formed between six conserved cysteine amino acid residues [119]. Based on the differences in disulfide-bonding modes, the defensins family can be divided into three major subtypes, α, β, and θ [120]. In fish, β-defensins were initially successfully isolated and identified from model fish such as *Danio rerio*, *Takifugu rubripes*, and *Tetraodon nigroviridis* [121]. Fish beta defensins show significant tissue distribution specificity in the body and are mainly enriched in the mucosal barrier system, including the respiratory epithelium, urogenital system, gastrointestinal tract, and other digestive system mucosa [122]. Studies have shown that these β-defensins distributed in specific cells and tissues play a key role in the host immune defense system. Studies have shown that these β-defensins distributed in specific cells and tissues play a key role in the host immune defense system, exhibiting a broad spectrum of biological activities [123]. With respect to antibacterial activity, β-defensins have been functionally characterized in multiple fish species. For instance, mandarin fish (*Siniperca chuatsi*), silver biddy (*Gerres filamentosus*), and blunt snout bream (*Megalobrama amblycephala*) have been reported to exhibit significant inhibitory effects on bacterial growth [124,125,126]. Notably, in grass carp (*Ctenopharyngodon idella*), β-defensins (CiBDs) are abundantly expressed in the skin and display strong antibacterial activity against fish bacterial pathogens [120]. Regarding antiviral functions, β-defensins also contribute to host defense against viral infections. For example, β-defensin derived from orange-spotted grouper (*Epinephelus coioides*) was shown to exert antiviral activity against Singapore grouper iridovirus (SGIV) and red-spotted grouper nervous necrosis virus (RGNNV) and to function as a molecular adjuvant that enhances protective immunity [127,128]. Collectively, these findings demonstrate that fish β-defensins serve as multifunctional immune effectors, playing roles in both antibacterial and antiviral defense.

Histone-derived antimicrobial peptides (HDAMPs) are a class of biologically active fragments produced by the protein hydrolysis of nucleosome histones. These peptides were initially isolated and identified from the skin mucus of catfish (*Ictalurus punctatus*), and their significant inhibitory properties against aquatic pathogenic bacteria and fungi were first confirmed [129]. Subsequent studies have successively discovered histone-derived peptides with broad-spectrum antimicrobial activity in various fish species such as *Sparus aurata*, *Dicentrachus labrax*, *Carassius auratus*, and *Danio rerio* [130,131,132]. These antimicrobial peptides have selective killing effects on prokaryotic cell membranes and are almost non-toxic to eukaryotic cells, making them important molecules for the development of new antimicrobial drugs [133]. Liang et al. identified two novel histone genes, TOMacroH2A2 and TOH2B, from *Trachinotus ovatus* through transcriptome analysis. The derived peptides (To.mh2a and To.h2b), designed and synthesized based on sequence features, exhibited excellent antibacterial activity and did not induce hemolytic reactions within the effective concentration range, providing a new solution for the treatment of bacterial diseases in *Trachinotus ovatus* [134].

Cathelicidin antimicrobial peptides have unique synthesis and activation mechanisms in the immune system of vertebrates. These peptides are initially stored in the secretory granules of neutrophils in the form of inactive precursors and are converted into mature peptides with biological activity through protein hydrolysis by elastase during the immune response [135]. For the first time, researchers have identified cathelicidin from nonmammalian sources in rainbow trout (*Oncorhynchus mykiss*), which greatly expands our understanding of the evolutionary distribution of this type of antimicrobial peptide [136].

Piscidins antimicrobial peptides lack cysteine residues and therefore do not rely on disulfide bonds to maintain structural stability [137]. The amino acid sequence length of this family of peptides varies significantly, ranging from 18 to 46 [138]. The N-terminal region contains highly conserved histidine and phenylalanine enrichment regions, while the C-terminal region exhibits sequence variability [139]. The mature peptides of piscidins exhibit a typical amphiphilic α-helix conformation, and their amphiphilic structure enhances their membrane penetration ability [140]. The species of the piscidins family exhibit strong biological activity against various microorganisms, including antiparasitic, antifungal, antiviral, and even anti-tumor activities. Another important characteristic is their strong salt tolerance [141]. In fish, the combination of hepcidin and piscidins regulates the infection of *Vibrio parahaemolyticus* in *Sparus aurata* [142]. Three novel piscidins (MSpiscidin-3) were identified from *Micropterus salmoides*, which exhibited induced expression patterns after bacterial infection in multiple organs and tissues of fish [143].

Antimicrobial peptides play a crucial role in the defense mechanisms of various organisms, exhibiting diverse biological activities like antibacterial, antifungal, antiviral, and even anti-tumor effects. Table 3 presents common antimicrobial peptides, along with their sources, structural features, and the targeted pathogens they act against, providing a concise overview of these important bioactive molecules.

## 3. Mechanism of Action

### 3.1. Direct Antibacterial Activity

The biological mechanism of antibacterial peptides (AMPs) is mainly reflected in the dual effect mode [20]: one is the direct microbial killing effect, and the other is the multifaceted immune regulatory function. The mechanisms of action of antimicrobial peptides are diverse, and different AMPs show specific antibacterial mechanisms [140]. According to the differences of action targets, direct antibacterial mechanisms can be further divided into two types: nonmembrane targeting and membrane targeting [148]. Nonmembrane targeting mechanisms include interfering with the synthesis of cell walls and inhibiting key metabolic processes in cells, and membrane targeting mechanisms destroy the integrity of microbial membranes [149]. The action specificity of antimicrobial peptides is regulated by multiple factors, including molecular characteristics (charge, hydrophobicity/hydrophilicity), structural parameters (chain length, secondary structure characteristics), and environmental factors (antimicrobial peptide concentration, targeted membrane lipids) [150]. Some AMPs can act through multiple mechanisms at the same time, and this multi-target property may be an important reason why they do not easily induce drug resistance.

**Figure 2:**
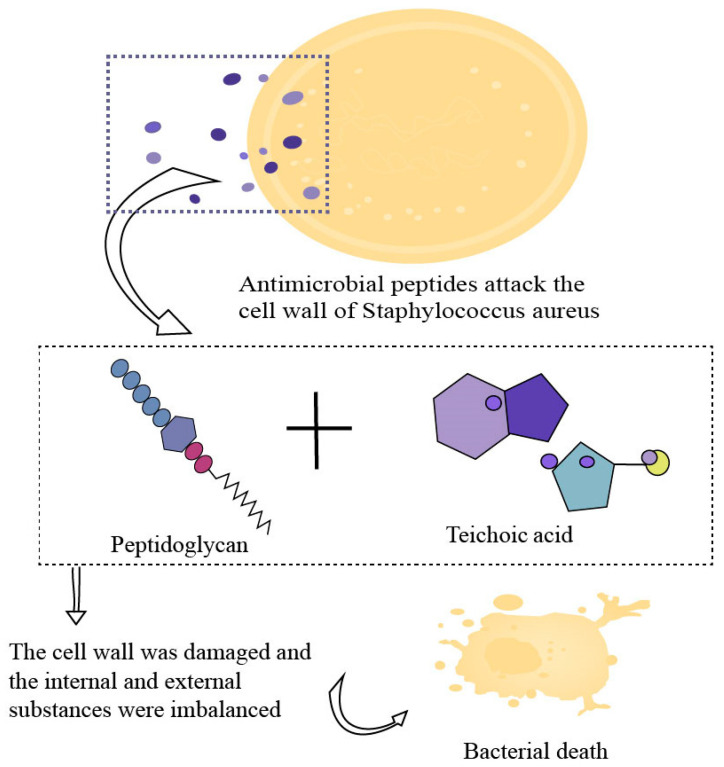
Mechanisms of cell wall injury. Taking *Staphylococcus aureus* as an example, AMPs directly act on the peptidoglycan and teichoic acid of the bacterial cell wall, destroying the cell wall structure and transmembrane potential, leading to osmotic imbalance and content leakage and ultimately to bacterial lysis and death. Created in by Fan Zhou (2025) https://BioRender.com. (accessed on 10 May 2025).

Cell wall targeting mechanism: AMPs act on bacterial cell walls through multiple pathways [151]. The core component of bacterial cell walls is peptidoglycan (PGN), whose synthesis process relies on the key precursor molecule lipid II, which is located on the cytoplasmic side of the cell membrane and serves as the basis for peptidoglycan chain extension and cross-linking [152]. Some AMPs (such as bacteriocins and vancomycin) can selectively recognize and bind to the pyrophosphate group of lipid II or the D-alanyl-D-alanine residue at the end of the peptide chain through specific functional groups in their molecular structure, hindering the elongation and cross-linking of peptidoglycan chains, ultimately leading to cell wall synthesis arrest [153]. Partial AMPs can directly act on key enzymes involved in peptidoglycan synthesis, inhibiting cell wall synthesis [154]. AMPs not only block synthesis but also disrupt existing cell wall structures by activating the bacteria’s own autolysis mechanism [155]. To better grasp the cell wall injury mechanisms of the AMPs described, refer to Figure 2, which, with *Staphylococcus aureus* as an example, vividly depicts the process of bacterial cell wall damage and subsequent death.

**Figure 3:**
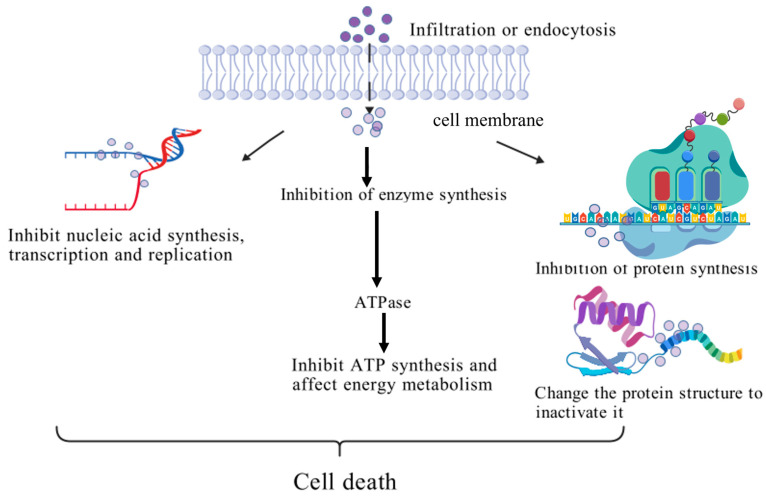
Mechanism of intracellular damage. Mechanisms of intracellular damage. Antimicrobial peptides enter bacterial cells through two primary pathways: (i) membrane penetration and pore formation, where AMPs interact electrostatically with negatively charged bacterial membranes and insert to form transmembrane channels, leading to membrane depolarization and content leakage; and (ii) transporter-mediated internalization, where certain non-lytic AMPs are actively translocated across the inner membrane via specific transporters. There are three main ways to cause bacterial death. First, they bind to nucleotides to inhibit the synthesis, transcription, and replication of nucleic acids. Second, AMPs can inhibit the activity of enzymes in bacterial cells, especially the activity of ATP synthase, which inhibits ATP synthesis and leads to energy metabolism damage. Third, AMPs can inhibit protein synthesis by blocking the normal functioning of ribosomes. Created in by Fan Zhou (2025) https://BioRender.com. (accessed on 10 May 2025).

Intracellular targeting mechanism: AMPs with intracellular targets exert antibacterial functions in a nonmembrane lytic manner, and their intracellular inhibitory activity exhibits diverse characteristics, covering multiple dimensions such as nucleic acid synthesis regulation, metabolic pathway interference, and cell division blockade [156]. AMPs break through the cell membrane barrier primarily through direct transmembrane permeation. In eukaryotic cells, certain AMPs may enter via endocytosis; however, bacteria lack the endocytic machinery, and thus bacterial entry relies on membrane disruption, pore formation, or energy-dependent translocation systems that facilitate peptide internalization without involving endocytosis [157]. After entering the cytoplasm, AMPs specifically recognize intracellular target molecules. Cheng et al. discussed and summarized AMPs with reported intracellular targeting activity and their intracellular target sites [158]. AMPs with different structures have differentiated targets and unique mechanisms of action [159]. In terms of nucleic acid metabolism regulation, some antimicrobial peptides can directly interact with DNA or RNA molecules, interfering with the replication, transcription, and translation processes of genetic information [160]. Haney et al. found that Puroindoline B-derived Peptide (PuroB) antimicrobial peptides can penetrate bacterial plasma membranes and block the biosynthesis of DNA, RNA, and proteins by binding to intracellular nucleic acids [161]. At the level of cell division regulation, AMPs achieve growth inhibition by inhibiting DNA replication and damage repair systems, and several fish-derived antimicrobial peptides inhibit bacterial cell division through intracellular targeting. For example, TFPI-1 C-terminal-derived peptide (26 aa) (TC26) from common carp (*Cyprinus carpio*) tissue factor pathway inhibitor 1 (TFPI-1) penetrates bacterial cells and induces the degradation of genomic DNA and RNA, while NK-Lysin-derived Peptide (27 aa) (NKLP27) from tongue sole (*Cynoglossus semilaevis*) NK-lysin induces the degradation of bacterial genomic DNA [162,163]. The 40 amino acid residue Mother Cell Inhibitor of the FtsZ (MciZ) peptide is an effective inhibitor of bacterial cell division, Z-ring formation, and localization [164]. AMPs can also block the process of nucleic acid damage repair by targeting DNA repair enzymes or interfering with repair signaling pathways [165]. Metabolic pathway interference is another important mechanism by which AMPs exert antibacterial activity. Some peptides can inhibit the activity of nucleic acid synthase, protein synthase, and cell wall synthesis-related enzymes while also acting on the cellular energy metabolism system by inhibiting ATP synthase activity or blocking electron transfer chains, cutting off the ATP generation pathway [166]. In terms of protein synthesis regulation, AMPs can directly destroy the spatial structure of proteins, leading to their loss of function [167], and can also inhibit translation processes by interfering with ribosome function [168]. Figure 3 graphically summarizes the intracellular damage mechanism of antimicrobial peptides, making the complex pathways of bacterial cell death more accessible.

**Figure 4:**
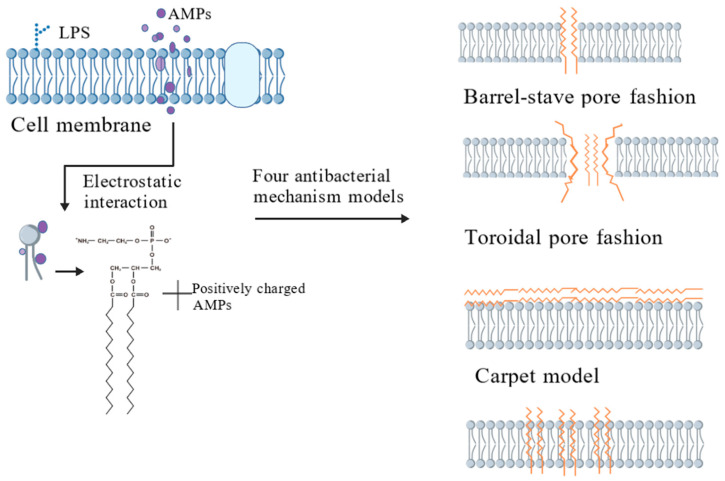
Mechanisms of cell membrane damage and four models of cell membrane targeting mechanisms. Positively charged AMPs interact with phospholipids to form channels and pores in the cell membrane, leading to the leakage of substances within the cell and ultimately resulting in cell death. The four mechanisms of action models include barrel-stave pore fashion, toroidal pore fashion, carpet model, and aggregation model. Created in by Fan Zhou (2025) https://BioRender.com. (accessed on 10 May 2025).

Membrane targeting mechanism: the receptor-independent membrane solubilization and bactericidal properties of AMPs have attracted much attention, and membrane interaction is the key link for them to exert direct antibacterial effect [169]. Based on the principle of charge complementarity, positively charged AMPs specifically bind to the negative charge sites on the surface of the bacterial cell membrane through electrostatic attraction. This initial interaction process lays the foundation for subsequent membrane destruction and intracellular penetration [170]. The main components of the cell membrane are lipids and proteins, and the phospholipid bilayer is used as its basic scaffold. With the help of Fourier transform infrared spectroscopy (FTIR) and molecular dynamics (MD) simulation technology, Cashman Kadri et al. systematically analyzed the mode of action of sjgap (bonito tuna GAPDH-related antimicrobial peptide) and its derivatives with the bacterial membrane model [171]. According to the difference in the cell envelope structure, bacteria are usually divided into two families: Gram-positive and Gram-negative. The former is wrapped with a thick layer of peptidoglycan outside the cell membrane, while the latter has a three-layer structure composed of an outer membrane, a thin layer of peptidoglycan, and an inner membrane [169]. Among them, lipopolysaccharide (LPS) of Gram-negative bacteria and lipoteichoic acid of Gram-positive bacteria carry high-density negative charges and become important recognition targets of AMPs [172]. Most AMPs achieve bactericidal functions through a series of processes such as electrostatic adsorption, membrane surface migration and aggregation, and membrane insertion [173]. The membrane perforation mechanism mainly covers two theoretical models, the transmembrane pore model and non-porous model. It includes two modes: the barrel and annular hole. In the barrel model, AMPs monomers first bind to the membrane surface, and the positively charged end of the peptide interacts with the phospholipid fatty acid chain hydrophobically and then vertically inserts into the lipid bilayer to form a through channel with the hydrophobic end outward and the hydrophilic end inward, causing the leakage of intracellular substances [174]. The annular pore model emphasizes that the peptide aggregation induces membrane depolarization, and the peptide lipid complex coils together to form micelle-like holes [175], leading to the efflux of biomacromolecules [176]. The non-porous model is represented by the carpet and polymerization mechanism. In the carpet model, AMPs form a two-dimensional array to cover the membrane surface. When the concentration exceeds the threshold, the peptide segment polymerizes into micelles to trigger membrane lipolysis [177]. The polymerization model describes the formation process of peptide phospholipid complexes, which insert into the membrane to form permeability channels, thereby promoting the intracellular transport of AMPs [178] Once in, these AMPs can exert intracellular targeting effects, including inhibiting nucleic acid synthesis, protein synthesis, and enzyme activity [179]. To offer a more intuitive insight into the aforementioned antibacterial mechanism models, Figure 4 graphically depicts the processes of cell membrane damage and the corresponding targeting mechanisms.

### 3.2. Immunomodulatory Effect

Antimicrobial peptides, as an important part of innate and adaptive immunity, play a key role in the process of resisting pathogen invasion and show high antibacterial and antiviral activities against bacteria, fungi, and viruses [180]. The lymphoid organs of fish are mainly composed of central lymphoid organs (responsible for the differentiation and maturation of immune cells, including thymus and head kidney) and peripheral lymphoid organs (responsible for initiating the immune response, including spleen and gill/gut/body surface-associated mucosal lymphoid tissues, and some fish also have lymph node like structures) [181]. The fish immune system covers two major systems, nonspecific innate immunity and specific adaptive immunity [182], and AMPs can strengthen the overall defense ability of the host by synergistically regulating these two immune responses [183].

The immunoregulatory functions of AMPs are diverse. In addition to directly killing pathogens, they can also mediate the recruitment and activation of immune cells, including macrophages, monocytes, dendritic cells, lymphocytes (CD4^+^ and CD8^+^ T cells, B cells), and natural killer cells (NK cells) [184]. At the molecular mechanism level, the expression regulation of AMP mainly depends on the signal pathway mediated by pattern recognition receptors (such as toll-like receptor TLR), or in response to the release of pro-inflammatory cytokines. For example, hepcidin regulates IL-1β and SOCS3 gene expression through the TNF signaling pathway [185]. In addition, AMPs can change the gene expression profile of host cells, induce the synthesis of chemokines and cytokines, promote the migration of leukocytes to the site of infection, affect the process of cell differentiation and activation, and participate in the activation or inhibition of the TLR signaling pathway [186].

**Figure 5:**
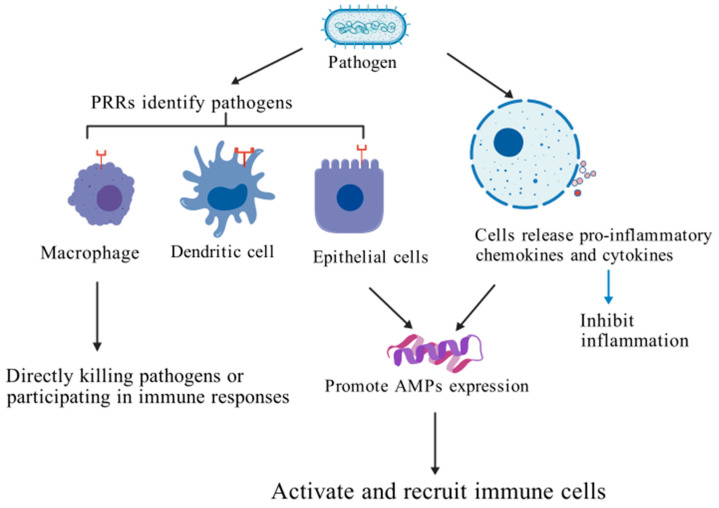
Immune regulatory mechanism. AMPs, as mediators of innate immunity, have the ability to directly kill pathogens and recruit and activate immune cells. Pathogens are recognized by pathogen recognition receptors (PRRs) such as macrophages, dendritic cells, and epithelial cells, and then removed through phagocytosis. They release pro-inflammatory chemokines and cytokines from cells, promote AMPs expression, and ultimately induce the recruitment and activation of other immune cells to the site of infection. Created in by Fan Zhou (2025) https://BioRender.com. (accessed on 10 May 2025).

In terms of inflammation regulation, AMPs exert anti-inflammatory effects through multiple pathways: on the one hand, they activate nuclear factor kappa B (NF-κB) and mitogen activated protein kinase (MAPK) pathways to achieve the bidirectional regulation of the inflammatory response [187]. On the other hand, it neutralizes pro-inflammatory cytokines released by macrophages and monocytes and alleviates excessive inflammatory injury [166]. At present, academia has proposed three models to explain the immune regulation mechanism of AMPs: in the “alternative ligand model”, AMPs directly bind to cell surface receptors to trigger signaling. In the “membrane disruption model”, AMPs indirectly regulate receptor activity by locally modifying the membrane structure. The “transactivation model” emphasizes that AMPs induce the release of membrane-bound factors, which in turn activates downstream signaling pathways. For a more intuitive understanding of how AMPs function in the immune regulation pathways described in this section, refer to the diagram (Figure 5) that outlines the key processes.

Studies have shown that AMPs do not rely on a single mode of action to function but show the characteristics of multi-mechanism synergy [188]. Its antibacterial process is often achieved through combination with multiple targets or the synergistic interaction between multiple peptide molecules, which breaks through the limitation of traditional single-target action [189]. This mechanism not only significantly improves the antibacterial efficacy of AMPs but also effectively delays the generation and spread of bacterial drug resistance [190].

## 4. Application in Aquaculture

### 4.1. Disease Prevention and Treatment

Aquaculture demands high water quality, with the prevention and control of infectious diseases being a crucial aspect of intensive production. Typically, high stocking densities and stressful conditions on fish farms make them highly susceptible to infections [191]. In recent years, biological control agents have gradually gained recognition as a means to avoid microecological imbalances in the aquaculture environment and improve water quality. AMPs derived from fish are one of the important sources of biological control agents for water quality improvement. The feed rich in AMPs can comprehensively improve the health level of aquatic animals [192]. It can enhance non-specific immunity (such as improving the activity of lysozyme [LSA], superoxide dismutase [SOD], and other enzymes), optimize the intestinal microbiota, improve antioxidant and disease resistance ability, and finally promote the growth performance and enhance the defense against pathogens [193]. In addition, AMPs as functional additives can also ensure feed safety from the source, effectively inhibit the proliferation of spoilage microorganisms in feed, and reduce the risk of biotoxin accumulation [184]. For example, SKL17-2 peptide from yellow croaker can specifically inhibit *Pseudomonas linguae*, can significantly reduce the incidence of visceral sarcoidosis, and is harmless to intestinal probiotics [194]. AMPs have the ability to inhibit the development of tumor cells or cause apoptosis. For example, synthetic fish AMPs (such as epinecidin-1 and TH2-3) can selectively inhibit the proliferation and migration and induce the apoptosis of tumor cells such as osteosarcoma, but they have low toxicity to normal cells [195]. In addition, some AMPs, such as cecropin, have immunomodulatory functions and can be used as potential immune adjuvants to enhance the immune response of protein antigens or subunit vaccines.

### 4.2. Preservation of Aquatic Products

Fish meat contains high levels of moisture, protein, and polyunsaturated fatty acids, making it highly perishable and susceptible to degradation by various biochemical, physical, and microbial factors throughout the manufacturing chain [196]. At present, chemical synthetic preservatives are widely used in the field of food preservation to extend the shelf life. However, a large number of studies have shown that such additives may cause multiple adverse reactions. Therefore, as people’s awareness of food safety increases, the application of natural, green, and efficient food preservatives has become mainstream [197]. Compared to chemical preservatives, AMPs can preserve food without altering its quality and are harmless.

In the field of aquatic products preservation, the application of lactic acid bacteria (LAB) and its metabolites (such as bacteriocins) as natural preservatives has multiple advantages, effectively improving the safety of food and delaying food corruption, inhibiting the generation of biogenic amines (such as histamine, cadaverine) and nitrogen-containing odor substances (such as Trimethylamine oxide) [198]. It is particularly noteworthy that natural antibacterial substances from aquatic sources have a significant inhibitory effect on foodborne pathogenic bacteria and common spoilage microorganisms [199,200]. For example, protamine has been shown to inhibit the growth of common spoilage bacteria, including *Pseudomonas* and *Shewanella*, and delay the production of spoilage markers such as total volatile basic nitrogen, thereby extending the shelf life of chilled fish fillets [101]. The application of antimicrobial peptides in aquatic products preservation mainly adopts two technical paths: one is the direct treatment process. In the processing link or cold chain storage and transportation process, the antimicrobial peptide solution is evenly distributed on the product surface by spraying or impregnation, which specifically inhibits dominant spoilage bacteria such as *Pseudomonas* and *Shewanella* and can extend the shelf life by 30–50% under the storage condition of 4°C [201,202]. Second, there is active packaging: antibacterial peptides are embedded in degradable packaging film to achieve slow-release antibacterial effects and reduce the risk of deterioration in cold chain transportation [203].

### 4.3. Antibiotic Alternatives

The traditional treatment of pathogens is highly dependent on antibiotic therapy [204]. However, due to their single target of action and the ability to significantly increase bacterial mutation frequency through mechanisms such as activating the bacterial SOS repair system and rpoS stress response pathway, these drugs inevitably lead to the emergence of drug-resistant strains during long-term widespread use [205]. AMPs are a superior alternative to current antimicrobials due to their novel mechanisms, potent activity, and effectiveness against drug-resistant infections. Compared to antibiotics, AMPs have significant advantages [206]. Research has shown that most AMPs exert their effects through non-specific multi-target mechanisms, mainly by interacting with bacterial surface components. This approach does not easily induce bacterial mutations directly and has a low incidence of drug resistance [207]. In tilapia culture system supplemented with cecropin and different antibiotics, there are significant differences in the distribution of antibiotic resistance genes (ARGs) in water, residue, and fish muscle samples, and cecropin as an antibiotic substitute may help reduce the spread of ARGs [208]. In addition, the rapid bactericidal properties of AMPs further reduce the risk of bacterial resistance [209]. AMPs exhibit broad-spectrum antibacterial activity [172], and also have significant inhibitory effects on various pathogens such as Gram-positive bacteria, Gram-negative bacteria, fungi, viruses, and protozoa [23]. It is worth noting that the combination of AMPs and traditional antibiotics can produce a synergistic effect, which not only enhances the penetration and absorption of antibiotics but also significantly improves the antibacterial effect [210]. For example, antimicrobial peptides K4 or K5 combined with traditional antibiotics can inhibit the development of the drug resistance of Gram-negative bacteria [211]. Mechanistic studies revealed that K4 and K5 exert their anti-resistance effects through two complementary pathways: (i) inhibition of drug efflux, which prevents antibiotic extrusion from bacterial cells; and (ii) enhancement of outer membrane permeability, where K4/K5 act as membrane disruptors to facilitate antibiotic entry into resistant bacteria [212]. As endogenous immune molecules involved in both innate and adaptive immunity, natural AMPs can be effectively hydrolyzed by digestive tract proteases due to their protein nature and can be completely degraded into amino acids in the human body. Under natural conditions, natural AMPs are easily decomposed by microbial enzymes and will not cause secondary problems related to pollution, bacterial drug resistance, or ecosystem damage [213,214,215].

## 5. Challenges and Limitations

### 5.1. Production Challenges

Although AMPs show great potential to replace traditional antibiotics, the following key factors still need to be considered in the process of clinical transformation. Compared to antibiotics, natural antimicrobial peptides often exhibit inferior efficacy, primarily due to their insufficient activity and stability under specific conditions, as well as the difficulty in precise regulation [216]. The large-scale commercial production of antimicrobial peptides faces two major difficulties: first, it is difficult to produce in large quantities directly from fish, which requires complex means such as biotechnology [11,12]. Therefore, developing efficient production technology and reducing costs are key to practical applications.

### 5.2. Environmental Stability

The physicochemical stability of AMPs is affected by a variety of environmental factors. Under high-salinity conditions, the electrostatic interaction will be reduced. The binding of specific AMPs (such as histostatin 5) to Fe^2+^ will lead to the unwinding of the α-helix structure [217]. Most AMPs remain stable in the pH range of 4.0–9.0, but individual AMPs are only effective in a narrow pH range [148]. The change in structure will affect the function. Replacing with tryptophan residues linked to hydrogen bonds can disrupt the charge distribution of the polar face of the helix, because the proportion of hydrophobic core changes affects the membrane selectivity [218]. Proteases have a strong destructive effect on AMPs, and pathogen proteases (such as CPAF) can specifically cleave host AMPs.

### 5.3. Drug Resistance Risk

AMPs still face several key challenges in their application, and it has been found that some pathogens can adapt through membrane lipid modification. Long-term exposure to sub-inhibitory concentrations can induce the formation of drug-resistant bacteria [219], and residues in the environment may alter the structure of microbial communities [220]. In addition, the dose–response relationship of AMPs is uncertain and lacks agreed-upon efficacy evaluation criteria.

## 6. Future Development Direction

### 6.1. Biotechnology Progress

Biotechnology can mine novel antimicrobial peptide sequences from a variety of organisms through genomics and metagenomics methods [221]. Machine learning tools (e.g., CalcAMP) can then predict activity, toxicity, and stability prior to synthesis, reducing experimental costs [222,223]. Candidate AMPs should be further enhanced through targeted modifications, including amino acid substitution to improve hydrophobicity or cationicity [224], chemical modifications (such as N-terminal acetylation/c-terminal amidation, unnatural amino acid substitution, selective halogenation, and cyclization modification), as well as deep learning for specificity optimization [225,226,227,228,229,230]. Production scale-up requires recombinant expression systems and CRISPR-mediated host engineering.

### 6.2. Carrier Innovation

AMPs are currently limited by protease degradation, poor absorption, and rapid in vivo clearance [231]. Nanoparticle carriers can protect AMPs from degradation and enhance mucosal adhesion [232,233]. The modified carrier function triggers the realization of targeted drug delivery at the infection site according to environmental changes [234]. Natural polymers offer biocompatibility, such as chitosan and alginate [235,236], while synthetic polymers provide controlled degradation, such as poly lactic-co-glycolic acid (PLGA), polyethylene glycol (PEG), and polycaprolactone (PCL) [214]. Beyond polymeric carriers, inorganic materials, metal nanoparticles, liposomes, and self-assembled systems, including WS_2_ quantum dots (2–5 nm), enhance AMPs stability, enable targeted delivery and controlled release, and offer additional functionalities such as fluorescence tracing and antimicrobial synergy [237,238].

### 6.3. Combined Application

AMP monotherapy may require high doses, and low-level tolerance has been observed [239]. As an emerging therapy, peptide drug conjugates (PDC) successfully overcome the drug resistance and improve the pharmacokinetic properties of vancomycin through the PDC series of vancomycin formed by coupling vancomycin with the highly basic lipidated membrane targeting peptide C-terminus [240]. The combination of antimicrobial peptides and antibiotics can produce synergistic effects, expand the antibacterial spectrum, and reduce the risk of bacterial resistance [241].

## 7. Conclusions

Antimicrobial peptides derived from fish represent a promising class of innate immune effectors that function through multiple mechanisms, including membrane disruption, intracellular targeting, and immune regulation, making them viable alternatives to traditional antibiotics in aquaculture. This article reviews the classification, tissue distribution, mechanism of action, and extraction and synthesis techniques, as well as practical applications of fish antimicrobial peptides. The latest advancements in recombinant expression systems, membrane-based purification, and peptide engineering have addressed key challenges in large-scale production, while nanoparticle encapsulation and stimulus-responsive delivery systems offer solutions for enhancing stability and oral bioavailability. In addition, the addition of AMPs to feed has been proven to enhance the growth performance, immune response, and disease resistance of various fish species. The synergistic combination of AMPs and antibiotics shows the potential to inhibit the development of drug resistance.

## Figures and Tables

**Table 1:** Extraction and purification methods, advantages, and disadvantages of antimicrobial peptides.

Method	Advantage	Disadvantage	References
Acid-base hydrolysis	Simple, economical, and efficient	The obtained AMPs have low purity, causing environmental damage	[67]
Proteolytic enzyme digestion	Mild conditions and cost savings	Enzymes exhibit specificity	[68]
Microbial fermentation	Proteases derived from microorganisms have diversity and can hydrolyze to produce peptide components with different molecular weights and amino acid sequences, and the cultivation conditions are economical and efficient.	Microbial fermentation has parameter fluctuations and low product purity.	[69,70]
Membrane filtration	Efficient purification and concentration, while maintaining its biological activity and integrity	The accumulation of liquid can lead to membrane fouling and blockage during continuous production processes	[71]
Chromatography	Excellent selectivity and resolution	The cost is high, and it requires the use of solvents harmful to the environment, posing a pollution risk.	[72]

**Table 2:** Synthesis methods and advantages and disadvantages of antimicrobial peptides.

Synthetic Method	Advantage	Disadvantage	References
SPPS	Expensive, consuming too much solvent	Faster speed, and automation is easy to implement	[99]
Block synthesis	More reagents need to be consumed	Synthesize longer oligomers	[100]
Enzymatic synthesis	Strict conditions, poor enzyme stability	The reaction conditions are mild, the enzyme exhibits high regional specificity, and there is no racemization	[101]
Recombinant synthesis	Both complex and expensive	Achieve high-level AMP expression, easier purification, and enhanced stability	[102]
Synthesis of antimicrobial peptides through genetic engineering	May produce cytotoxicity and be easily degraded by proteases	Optimize the design of gene coding sequences using biological and computational methods	[103]

**Table 3:** Common antimicrobial peptides and their sources, structures, and targeted pathogens.

Key Antimicrobial Peptides	Source	Structure	Biological Activities	References
Bolespleenin_334–347_	*Boleophthalmus pectinirostris*	Only 14 amino acid residues	Gram-negative bacteria	[144]
Epi-1	*Epinephelus coioides*	A peptide consisting of 21 amino acids	Methicillin-resistant *Staphylococcus aureus* (MRSA)	[145]
Larimicin_78–102_	*Larimichthys crocea*	The mature peptide consists of 249 amino acids	*Vibrio fluvialis*	[5]
piscidin 1	Mast cells in hybrid striped bass	The linear AMP, consisting of 22 amino acids, possesses an amino terminal rich in phenylalanine	Gram-negative pathogen	[138]
hepcidin	*Salmo salar*	The average length is 41 amino acid residues	Copepod ectoparasite *Caligus rogercresseyi*	[146]
LEAP-2	*Paralichthys olivaceus* *Oncorhynchus mykiss*	Two disulfide bonds formed by four highly conserved cysteine residues	Gram-positive bacteria (*Bacillus subtilis*, *Streptococcus agalactiae*, and *Lactococcus gasseri*) and Gram-negative bacteria (*Vibrio harveyi* and *Escherichia coli*)	[110]
LEAP-1	Tilapia	Containing four disulfide bonds, it can stabilize the β-pleated sheet structure formed by eight conserved cysteine residues	Anti-cancer properties	[147]
*Cm*Def	*Carangoides malabaricus*	63 amino acids	*Proteolytic vibrio* *Aspergillus hydrophila*	[119]

## Data Availability

No data are available due to privacy or ethical restrictions.

## Abbreviations

AMPs	Antimicrobial Peptides
APD	Antimicrobial Peptide Database
ARBs	antibiotic-resistant bacteria
Arg	arginine
ARGs	antibiotic-resistant gene
B cells	B lymphocytes
CalcAMP	Calculated Antimicrobial Peptide
CD4^+^ T cells	Cluster of Differentiation 4-positive T lymphocytes
CD8^+^ T cells	Cluster of Differentiation 8-positive T lymphocytes
CiBDs	*Ctenopharyngodon idella* β-Defensins
CRISPR	Clustered Regularly Interspaced Short Palindromic Repeats
*E. coli*	*Escherichia coli*
FTIR	infrared spectroscopy
GALT	gut-associated lymphoid tissue
GAPDH	Glyceraldehyde-3-Phosphate Dehydrogenase
GFC	gel filtration chromatography
HDAMPs	histone-derived antimicrobial peptides
HIC	hydrophobic interaction chromatography
IEC	ion exchange chromatography
IL-1β	Interleukin-1 Beta
LAB	lactic acid bacteria
LEAPs	Liver-expressed antimicrobial peptides
LPS	lipopolysaccharide
LSA	lysozyme
Lys	lysine
MAPK	mitogen activated protein kinase
MciZ	Mother Cell Inhibitor of FtsZ
MD	molecular dynamics
MRSA	Methicillin-resistant *Staphylococcus aureus*
MWCO	molecular weight cut-off
NF-κB	nuclear factor kappa B
NK cells	natural killer cells
NKLP27	NK-Lysin-derived Peptide (27 aa)
PCL	polycaprolactone
PCR	polymerase chain reaction
PDC	peptide drug conjugates
PEG	polyethylene glyco
PES	polyethersulfone
PGN	peptidoglycan
PLGA	poly lactic-co-glycolic acid
PRRs	pathogen recognition receptors
PuroB	Puroindoline B-derived Peptide
RGNNV	red-spotted grouper nervous necrosis virus
RP-HPLC	phase high performance liquid chromatography
SGIV	Singapore grouper iridovirus
SKL17-2 peptide	Synthesized Killer Peptide 17-2
SOCS3	Suppressor of Cytokine Signaling 3
SOD	superoxide dismutase
SPPS	solid-phase peptide synthesis
TC26 TFPI-1	C-terminal-derived peptide (26 aa)
TFPI-1	tissue factor pathway inhibitor 1
TH2-3	Tilapia Hepcidin 2-3
TLR	toll-like receptor
TNF	Tumor Necrosis Factor
TOH2B	Trachinotus ovatus Histone H2B
WS_2_	Tungsten Disulfide
